# Modelling the tail-phase pharmacokinetics of long-acting cabotegravir and rilpivirine from early pregnancy to postpartum at steady state

**DOI:** 10.1007/s10928-026-10065-4

**Published:** 2026-09-23

**Authors:** Shakir Atoyebi, Catriona Waitt, Adeniyi Olagunju

**Affiliations:** 1 Department of Biochemistry, Cell and Systems Biology, University of Liverpool, Biosciences Building, Crown Street, Liverpool, L69 7BE UK; 2 Centre of Excellence for Long-acting Therapeutics, University of Liverpool, Liverpool, UK; 3 Department of Women’s and Children’s Health, University of Liverpool, Liverpool, UK; 4 Infectious Disease Institute, Makerere University College of Health Sciences, Kampala, Uganda

**Keywords:** Cabotegravir, Rilpivirine, Long-acting, Pregnancy, PBPK, Tail-phase pharmacokinetics

## Abstract

Long-acting cabotegravir and rilpivirine combination (LA-CAB/RPV) is approved for HIV treatment whilst LA-CAB alone is approved for HIV prevention, both in adults. However, individuals who become pregnant might prefer to discontinue it due to lack of definitive data on safety. The aim of this study was to characterise the tail-phase maternal and fetal pharmacokinetics of LA-CAB/RPV following discontinuation at steady-state early in pregnancy using physiologically-based pharmacokinetic modelling. A virtual population of non-pregnant women (*n* = 100 per scenario) initiated intramuscular injections of LA-CAB/RPV at the approved dosage and continued maintenance dose (400/600 mg once monthly or 600/900 mg once every two months) until steady state. We simulated discontinuation at steady state after only one injection during pregnancy. Tail-phase pharmacokinetics of CAB and RPV from LA injections were characterised during gestation and until 64 weeks postmenstrual age. Pharmacokinetic tails of LA-CAB/RPV were driven by the residual drug in the muscle depot which stabilised at steady state and reduced steadily upon dosing discontinuation. Upon discontinuation of the monthly dosing, predicted median (IQR) maternal plasma concentrations for LA-CAB were 415 (386–448) ng/mL at delivery and 125 (115–139) ng/mL at 64 weeks postmenstrual age. For LA-RPV, these were 11.6 (11.0-12.6) ng/mL and 7.84 (7.30–8.49) ng/mL at delivery and at 64 weeks postmenstrual age, respectively. Pharmacokinetic tails of LA-CAB/RPV extend to several months postpartum, with levels falling below established minimum effective concentration in most women after gestation week 33. Potential strategies to minimise potential risks associated with LA-CAB/RPV discontinuation in this population are needed.

**Graphical Abstract:**

**Figure:**
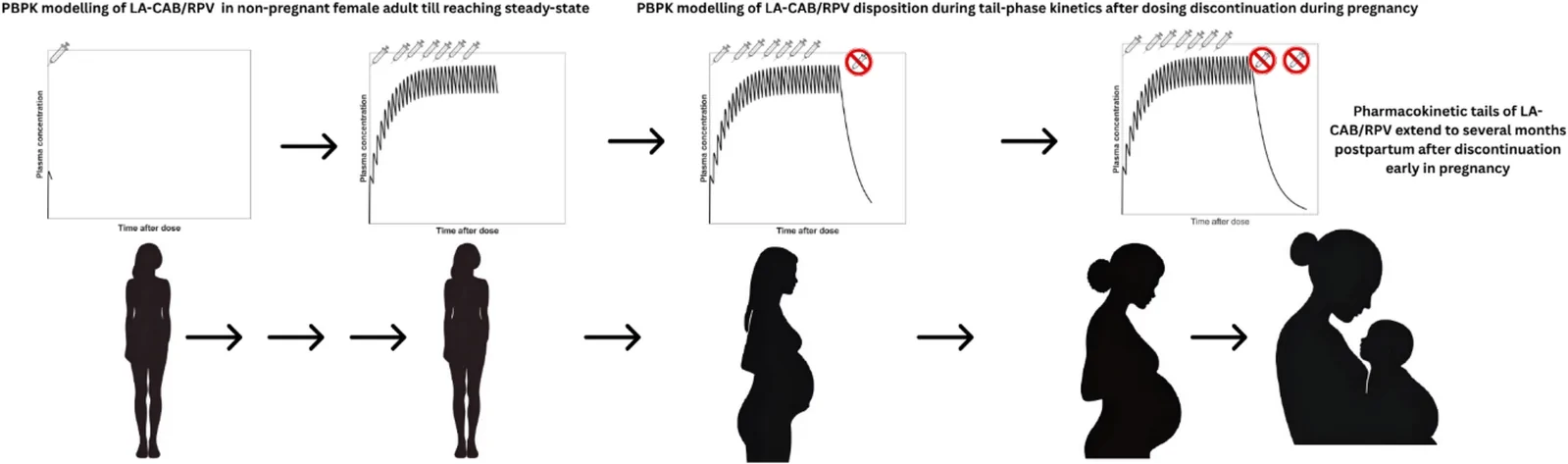

**Supplementary Information:**

The online version contains supplementary material available at https://doi.org/10.1007/s10928-026-10065-4.

## Introduction

Over 21 million women and girls were recently estimated to be living with HIV which amounts to 53% of the global burden of HIV [1]. Antiretroviral therapy has improved the life expectancy and quality of life of people living with HIV; an estimated 83% of women aged 15 years and above had access to HIV treatment in 2024 [1–3]. Despite the benefits of antiretroviral therapy, some factors affecting drug adherence limit the use of oral antiretroviral drugs for optimal prevention and treatment of HIV. These factors include dosing frequency, pill burden, concerns about disclosure, and fear of stigma among others [4, 5]. Moreover, poor drug adherence risks virologic failure, HIV transmission and the development of drug resistance [4, 6]. Long-acting injectable (LAI) antiretroviral options offer relief from daily adherence barriers but create a prolonged tail-phase after discontinuation, during which plasma concentrations decline slowly and may become subtherapeutic [7, 8]. Long-acting injectable cabotegravir and rilpivirine combination (LA-CAB/RPV) is licensed as a complete regimen for the treatment of HIV-1 infection in adults who are virologically suppressed with every 4 weeks (Q4W) or every 8 weeks (Q8W) dosing after an optional oral lead-in [9, 10].

Tail-phase of long-acting drugs could be defined as the prolonged post discontinuation interval with declining, sometimes subtherapeutic plasma levels due to slow depot release and long apparent half-lives. Landovitz et al. [8] reported LA-CAB remained detectable in the body 76 weeks after dosing discontinuation in some individuals. Further analyses suggested that LA-CAB could remain detectable in the body for up to 3 years depending on sex and body mass index (BMI) of the individual [8]. For many anti-infective drugs, drug concentrations in the body must be kept above certain clinical thresholds to maintain effect which led to commonly used indices like multiples of the protein-adjusted drug inhibitory drug concentration. Thus, there could be an increased drug resistance risk if concentrations hover below efficacy targets for weeks to months during the tail-phase without the introduction of other suitable antiretroviral drugs.

HIV can be transmitted during pregnancy, at delivery and during breastfeeding [11, 12], but antiretroviral therapy could reduce the risk of vertical HIV transmission to < 1% [12–14]. In 2024, 84% of pregnant women living with HIV had access to antiretroviral treatment to prevent vertical transmission of HIV to their child [1]. However, pregnancy brings multiple physiological changes which can affect drug disposition [9, 15]. Cabotegravir and rilpirivirine are substrates of UGT1A1 and CYP3A4, respectively. Thus, pregnancy-associated increases in UGT1A1 and CYP3A activity, expanded plasma volume, and reduced protein binding may reduce drug exposures with uncertain implications for depot driven tail concentrations of both cabotegravir and rilpivirine [16–18]. Notably, reduction in rilpivirine exposure has been predicted for LA-RPV initiated during pregnancy which raises concerns about maintaining efficacy during pregnancy [19]. In addition, the fetus is exposed to maternal antiretroviral drugs taken during pregnancy which potentially serves as pre-exposure prophylaxis before delivery [20].

Nonetheless, due to lack of definitive data on safety (and efficacy), there are few reports of women who discontinued LA-CAB/RPV for antiretroviral treatment because they were planning to become pregnant [21]. Also, LA-CAB/RPV was discontinued in some women during a clinical study when they became pregnant [22]. For similar reasons, some women who become pregnant may wish to discontinue LA-CAB for PrEP or LA-CAB/RPV for antiretroviral treatment during gestation. However, LA-CAB/RPV has a long wash-out period and drug concentrations of cabotegravir and rilpivirine might persist in the body system beyond the period of gestation to lactation when stopped close to the conception period [22]. Thus, though LAI administration could be stopped shortly before or early in pregnancy, the developing embryo/fetus would likely be exposed to the drug being released from the muscle depot throughout gestation. This exposure might also persist after the delivery of the baby with potential drug exposure during lactation.

Physiologically-based pharmacokinetic (PBPK) modelling is a computational tool increasingly used during drug research and development to predict drug disposition and guide related clinical studies [23]. PBPK models can facilitate exploration of dose adjustments in complicated clinical scenarios like drug-drug interactions, renal and hepatic impairment [24–26]. In addition, they are often employed to predict drug disposition in specific populations like pregnant women and paediatrics [27, 28]. More recently, we have used PBPK models to predict the disposition of long-acting antiretroviral and antipsychotic drugs during pregnancy [9, 29]. In our prior modelling study, LA-CAB/RPV was initiated at different trimesters during pregnancy depicting clinical scenarios without earlier drug exposure before pregnancy. Here, we model discontinuation at early pregnancy after pre-conception steady state—quantifying maternal, fetal, and postpartum tail exposures by regimen. Currently, no study has quantified both the mother–fetus ‘tail-exposures’ after stopping at early pregnancy once steady state is established preconception, across Q4W and every two months regimens. Though Patel et al [22] reported the tail exposures of LA-CAB/RPV in the mother after discontinuing, fetal exposure during this phase was not reported [22]. Also, many of the participants did not reach steady state at the time of dosing discontinuation. In addition, some of the study participants were switched to drugs known to induce or inhibit enzymes that metabolise cabotegravir or rilpivirine which could have impacted their disposition during the tail phase. If drug concentrations during the tail-phase fall below minimum effective concentrations for sustained periods, the risk of virologic rebound and resistance selection would increase. Thus, subtherapeutic drug concentrations during the tail-phase could compromise virologic suppression and select drug resistance in treatment settings unless a suitable oral regimen is introduced [21]. Prolonged low levels of rilpivirine increase the risk of drug resistance to non-nucleoside reverse transcriptase inhibitors. Likewise, fetal exposure during gestation to subtherapeutic drug concentrations also poses uncertain benefit/risk.

In this study, we aimed to (1) quantify maternal tail pharmacokinetics of cabotegravir and rilpivirine after discontinuation at early pregnancy following preconception steady state, and (2) predict fetal and postpartum exposure. Clinically relevant metrics such as time to subtherapeutic levels and cord-to-maternal ratios were evaluated to inform decisions on counselling and timing of the administration of an alternative oral regimen.

## Methods

The development and validation of the adult and pregnancy PBPK models used for this study have been reported previously [9]. Because the present analysis applies those models to prediction of tail-phase exposure after discontinuation in early pregnancy, we summarise here the clinical datasets that informed model development and/or evaluation. The framework was supported using oral cabotegravir, rilpivirine and raltegravir data in non-pregnant adults; LA-CAB/RPV datasets in non-pregnant adults; oral rilpivirine and raltegravir data during pregnancy; and clinical washout data after discontinuation of LA-CAB/RPV early in pregnancy [9, 22, 30–38].

Due to limited pregnancy clinical pharmacokinetic data for cabotegravir, raltegravir was retained as a probe substrate during pregnancy model development because both compounds are metabolised by UGT1A1 and UGT1A9. This allowed assessment of whether the model adequately represented pregnancy-associated changes relevant to UGT-mediated disposition. Relevant drug input parameters, describing pregnancy-induced physiological changes, and model qualification outputs are provided in Online Resource Tables S1-S5 [9]. Given the scarcity of pregnancy-specific long-acting clinical data, the present simulations should be interpreted as fit-for-purpose model-informed projections rather than direct substitutes for observed clinical pharmacokinetic datasets.

**Fig. 1:**
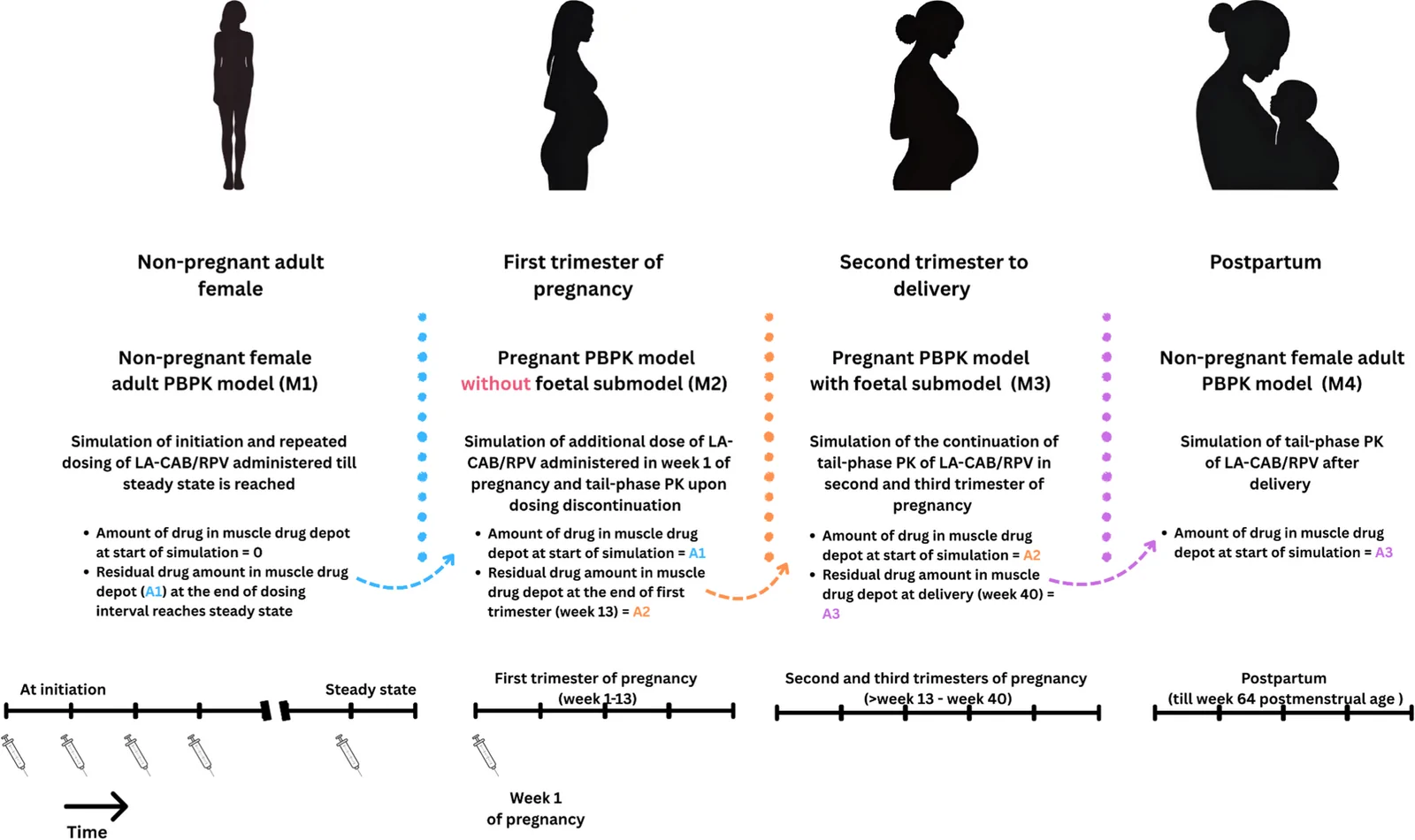
Modelling workflow for simulations for tail-phase pharmacokinetics of LA-CAB/RPV discontinued early in pregnancy when steady state is reached before conception. The figure illustrates the different populations, distinct model types used, and their related simulations. The dosing regimen at initiation to steady state was 600 mg LA-CAB followed by 400 mg LA-CAB every 4 weeks for cabotegravir and 900 mg LA-RPV followed by 600 mg LA-RPV every 4 weeks for rilpivirine. Subsequently, 600 mg LA-CAB and 900 mg LA-RPV were administered at the first week of pregnancy

The model was utilised on SimBiology (MATLAB, R2019a). This current study explored the tail-phase kinetics of LA-CAB/RPV if discontinued in early pregnancy after steady state has been reached before conception. As illustrated in Fig. 1, model simulations were conducted through 3 different conditions: before, during and after pregnancy (i.e. postpartum). An adult female PBPK model was used to simulate the non-pregnant populations before pregnancy and again after delivery (Fig. 1), using previously reported demographic and organ-weight parameters for a Caucasian population [39]. Two distinct pregnancy PBPK models were used during gestation. A pregnancy PBPK model without a fetal sub-model was used during the first trimester (up to gestational week 13) to characterise maternal disposition. A pregnancy PBPK model with a fetal sub-model was then used from gestational week 13 to delivery to characterise both maternal pharmacokinetics and fetal exposure. This segmented workflow was necessary because the pregnancy PBPK model with fetal sub-model could not accommodate gestational age values below week 13. Placental transfer was represented as bi-directional passive diffusion as previously described in literature [9, 40]. Fetal exposure estimates were generated only during the interval in which the fetal sub-model was operational (gestational week 13 to delivery).

To preserve continuity between model stages, the final residual drug amount in the intramuscular depot from one stage was carried forward as the initial depot amount in the next stage. This was considered an appropriate bridging strategy because the terminal decline after discontinuation is driven primarily by the slowly decreasing depot amount, which acts as the dominant rate-limiting source term during the tail phase. Maternal exposure was therefore linked across stages through preservation of the principal determinant of ongoing systemic input after dosing cessation. However, because separate model structures were used across stages, this workflow remains a source of model uncertainty and is acknowledged as a limitation below.

Approved dosing of cabotegravir and rilpivirine could start with an optional 1-month daily oral lead-in with 30 mg cabotegravir and 25 mg rilpivirine taken once daily. After the oral lead-in or without it, LA-CAB/RPV dosing starts with initial intramuscular (IM) injections of 600 mg LA-CAB and 900 mg LA-RPV at separate gluteal sites during the same visit. Thereafter, the IM dosing continues with 400/600 mg Q4W dosing of LA-CAB/RPV or Q8W dosing with 600/900 mg [41]. Similarly, LA-CAB administered alone (without LA-RPV) has been approved for HIV pre-exposure prophylaxis (PrEP) with its dosing being identical to the Q8W dosing of LA-CAB when administered for treatment [42].

As previously reported, drug release from the IM depot compartment was modelled as a first-order reaction shown in the Eq. (1) [9, 43]. The drug release rates were fitted in the model using available clinical data [30, 44]. An assumption was made that the drug release rate was not affected by pregnancy. Hence, the drug release rate determined in the non-pregnant adult PBPK model was also implemented in the pregnancy PBPK models.

1 $$\:\raisebox{1ex}{${A}_{muscle}$}\!\left/\:\!\raisebox{-1ex}{$dt$}\right.=\:{-K}_{IM}\times\:{A}_{IM\:depot,\:muscle}$$

where K_IM,_ is the release rate of drug (hr^− 1^) from the IM depot; A_IM depot, muscle_, is the amount of the drug (mg) in the IM depot within the muscle; and A_muscle_/dt, is the amount of drug released from the IM depot into the systemic circulation per time (mg/hr).

The dosing administration protocol employed in the model was based on the approved dosing of LA-CAB/RPV without the optional oral lead-in dose described above because reports have shown comparable drug levels with or without the oral lead-in for LA-CAB/RPV [45]. Thus, LA-CAB/RPV, without oral lead-in, was initiated and continued to steady state in a virtual population of 100 non-pregnant women. Dosing was continued till steady state was achieved in the non-pregnant state, continued through the period of conception and terminated in early pregnancy in the virtual cohort of 100 women. Apart from biological changes driven by pregnancy in the model, other baseline characteristics of the virtual participants were maintained across model stages. The last dose was administered in week 1 of pregnancy to reflect a clinically plausible situation in which an individual may receive one injection during early unrecognised pregnancy before discontinuation. Body weight increased dynamically during pregnancy according to the implemented gestational functions (Table S2). Simulations of the tail-phase pharmacokinetics of LA-CAB/RPV were continued in virtual populations of pregnant women from second trimester until delivery, and from delivery to 24 weeks postpartum (equivalent to 64 weeks postmenstrual age, assuming delivery at 40 weeks gestation). Continuous simulation of drug disposition from pre-pregnancy to the postpartum period was enabled via transfer of the final residual drug in muscle depot to the next stage (Fig. 1). Both maternal and fetal exposure measures were computed from concentration-time data for pregnancy (weeks 15–40) and postpartum. Maternal plasma concentrations during pregnancy and postpartum were compared against a value (or approximate value of) four times the protein binding-adjusted 90% inhibitory concentration (PA-IC_90_ = 166 ng/mL; 4*PA-IC_90_ = 664 ng/mL for cabotegravir & PA-IC_90_ = 12 ng/mL; ~4*PA-IC_90_ = 50 ng/mL for rilpivirine), which is often accepted as providing therapeutic plasma concentrations (i.e. clinical target concentrations) [46]. In addition, the time for the plasma drug concentration to reach below the clinical target concentrations after the last dose was also computed.

## Results

As reported previously, simulated PK metrics for oral cabotegravir, rilpivirine and raltegravir in adults, as well as for LA-CAB/RPV in adults, were generally within 2-fold of observed clinical values during model qualification [9]. In addition, for the pregnancy discontinuation setting most closely related to the present application, simulated terminal half-lives after LA-CAB/RPV discontinuation were within 2-fold of observations from Patel et al.; specifically, the observed-to-predicted ratios were 1.12 for CAB and 1.21 for RPV (Table S5) [9, 22]. Some comparisons approached the 2-fold boundary, indicating that while the framework was considered adequate for the present fit-for-purpose application, absolute exposure estimates should be interpreted with appropriate caution [9].

A total of 4 scenarios were simulated (*n* = 100) for each LA dosing regimen in virtual cohorts of non-pregnant, pregnant and postpartum women (Fig. 1). The median (interquartile range) age was 26.5 years (22.2–30.8) for all virtual cohorts. Also, the median (interquartile range) body weight for the pregnant and postpartum cohort was 64.1 kg (54.8–73.8) and 59.9 kg (51.5–68.5), respectively.

**Fig. 2:**
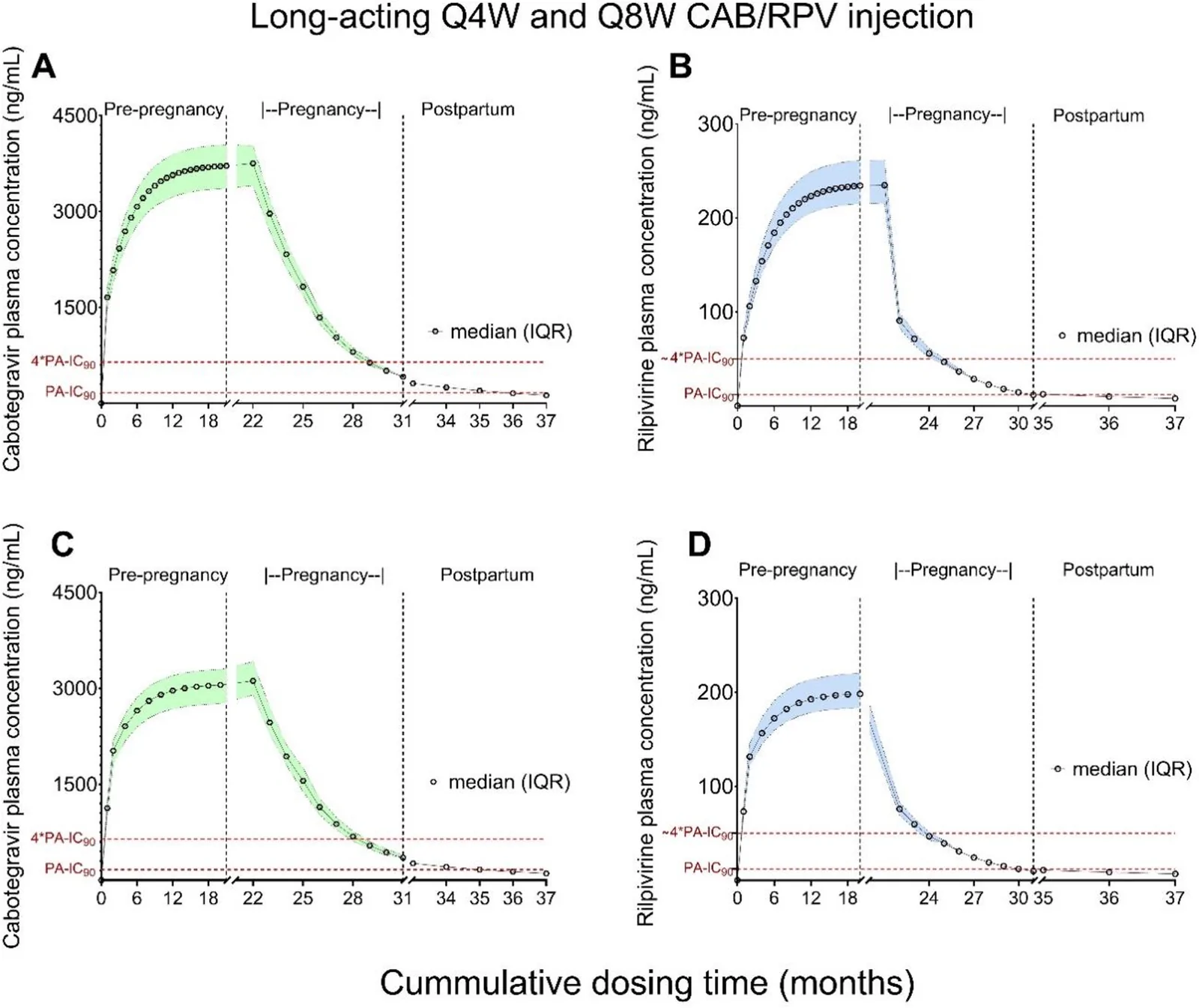
Predicted tail‑phase pharmacokinetics of monthly (Q4W) and bimonthly (Q8W) long‑acting cabotegravir/rilpivirine (LA‑CAB/RPV) from pre‑conception to 6 months postpartum. Panels show (**a**) Q4W cabotegravir (CAB); (**b**) Q4W rilpivirine (RPV); (**c**) Q8W cabotegravir and (**d**) Q8W rilpivirine total plasma concentrations (ng/mL). Curves depict the median with shaded bands indicating the interquartile range (IQR) across virtual participants (*N* = 100 per scenario). Vertical reference lines mark conception and delivery; background shading indicates pre‑conception dosing to steady state, pregnancy tail (no further injections), and postpartum tail. Horizontal dashed lines denote protein‑adjusted inhibitory thresholds: PA‑IC_90_ (CAB: 166 ng/mL; RPV: 12 ng/mL, respectively), 4×PA‑IC_90_ (CAB: 664 ng/mL), and ~ 4×PA‑IC_90_ (RPV: 50 ng/mL). Abbreviations: CAB, cabotegravir; RPV, rilpivirine; Q4W, every 4 weeks; IQR, interquartile range; PA‑IC_90_, protein‑adjusted 90% inhibitory concentration

The pharmacokinetic tails for LA-CAB and LA-RPV were driven by the residual drug in the muscle depot which stabilised at steady state (Fig. 2). Figure 2 shows the predicted plasma concentration profiles with the simulation of the tail-phase pharmacokinetics for the LA-CAB/RPV for both Q4W and Q8W dosing. With the Q4W dosing, the simulated residual drug in the muscle depot was 1546 mg and 2309 mg at steady state before pregnancy, 925 mg and 1383 mg at the end of the first trimester, and 208 mg and 312 mg at the end of pregnancy (week 40), for cabotegravir and rilpivirine, respectively. With the Q8W dosing, the simulated residual drug in the muscle depot was 1028 mg and 1542 mg at steady state before pregnancy, 774 mg and 1161 mg at the end of the first trimester, and 174 mg and 262 mg at the end of pregnancy, for cabotegravir and rilpivirine, respectively. These findings support the interpretation that the residual intramuscular depot is the principal driver of ongoing maternal exposure after discontinuation and therefore the key state variable linking successive model stages.

At gestation week 8, maternal plasma concentrations were predicted to exceed the clinical target concentrations (cabotegravir: 664 ng/mL; rilpivirine: ~50 ng/mL) in 100% of the cohort for all scenarios except Q8W LA-RPV, for which 98% remained above target; by delivery, 0% of participants were predicted to remain above these targets (Table 1). For LA-CAB, cabotegravir plasma concentrations were predicted to fall below the 4*PA-IC_90_ at a median (range) time of 30.1 weeks (24.4–36.1) and 27.4 weeks (13.1–32.7) after the last dose for Q4W and Q8W, respectively, during pregnancy. In addition, cabotegravir plasma concentrations were predicted to fall below the PA-IC_90_ at a median (range) time of 58.0 weeks (52.7–63) and 55.1 weeks (48.6–61.3) after the last dose for Q4W and Q8W, respectively, which occurs postpartum. With LA-RPV, rilpivirine plasma concentrations were predicted to fall below the ~ 4*PA-IC_90_ at a median (range) time of 13.3 weeks (9.00–18.7) and 11 weeks (6.14–14.0) after the last dose for Q4W and Q8W, respectively, during pregnancy.

**Table 1:** Predicted proportion of women with plasma drug concentration above the minimum effective concentration

	Q4W		Q8W	
	CAB	RPV	CAB	RPV
% Above 4*PA-IC_**90**_^**#**^				
Gestation week 8	100	100	100	98
At delivery (40 weeks)	0	0	0	0
**% Above PA-IC**_**90**_				
At delivery (40 weeks)	100	38	100	5
20 weeks postpartum	33	7	0	0
24 weeks postpartum	4	2	0	0

*CAB* – cabotegravir; *GW* – gestation week; *MEC* – minimum effective concentration; *PA-IC*_*90*_ – protein binding-adjusted 90% inhibitory concentration (166 ng/mL for cabotegravir and 12 ng/mL for rilpivirine); *Q4W* – every 4 weeks dosing; *Q8W* – every 8 weeks dosing; *RPV* – rilpivirine

^#^ ~4*PA-IC_90_ (50 ng/mL) was used for rilpivirine

Predicted cord-maternal blood ratio was greater than 1 for cabotegravir, but less than 1 for rilpivirine close to delivery (Table 2). Whilst predicted first quartile cord plasma levels were above 4*PA-IC_90_ for cabotegravir, predicted third quartile cord plasma levels were below PA-IC_90_ for rilpivirine (Table 2). Likewise, subtherapeutic plasma concentrations of cabotegravir might persist for a considerable period in the mother during lactation (Table 3).

**Table 2:** Predicted foetal exposure during the tail-phase pharmacokinetics of LA-CAB/RPV in pregnancy

Gestation age	Q4W		Q8W	
	CAB	RPV	CAB	RPV
**Cord plasma concentration (ng/mL)**				
Week 15	132 (123–140)	0.80 (0.75–0.86)	112 (103–123)	0.67 (0.63–0.72)
Week 27	1432 (1342–1564)	13.7 (13.0–14.8)	1220 (1105–1357)	11.5 (10.9–12.4)
Week 38	837 (786–914)	11.0 (10.4–11.8)	715 (648–792)	9.20 (8.73–9.91)
Week 40	764 (717–834)	10.2 (9.7–11.0)	652 (592–723)	8.56 (8.12–9.22)
**Cord-maternal** **blood ratio**				
Week 15	0.07	0.02	0.07	0.02
Week 27	1.69	0.57	1.69	0.57
Week 38	1.84	0.85	1.84	0.85
Week 40	1.85	0.88	1.85	0.88

Concentration data presented as median (interquartile range). Abbreviations: CAB – cabotegravir; Q4W – every 4 weeks dosing; Q8W – every 8 weeks dosing; RPV – rilpivirine

**Table 3:** Predicted maternal concentrations of LA-CAB/RPV at delivery and postpartum

Maternal plasma concentration (ng/mL)	Q4W		Q8W	
	CAB	RPV	CAB	RPV
At delivery	415 (386–448)	11.6 (11.0–12.6)	353 (318–393)	9.80 (9.21–10.6)
24 weeks postpartum	125 (115–139)	7.84 (7.30–8.49)	105 (96.0–116)	6.82 (6.30–7.42)

Concentration data presented as median (interquartile range). Abbreviations: CAB – cabotegravir; Q4W – every 4 weeks dosing; Q8W – every 8 weeks dosing; RPV – rilpivirine

Although second- and third-trimester simulations were implemented as one continuous gestational-age-dependent run from week 13 to delivery, outputs were extracted at prespecified gestational ages to aid interpretation of the evolution of fetal and maternal exposure across later pregnancy.

## Discussion

In this study, we applied a previously developed PBPK framework to a clinically relevant but understudied scenario: discontinuation of LA-CAB/RPV in early pregnancy after preconception steady state had already been achieved. For this use case, the principal methodological challenge is that exposure after discontinuation is governed not by additional dosing but by prolonged release from the residual intramuscular depot. The current analysis therefore extends prior pregnancy PBPK work by quantifying how that depot-driven tail may sustain maternal and fetal exposure through gestation and into the postpartum period. For the first time, predicted fetal exposure to LA-CAB/RPV during this tail-phase kinetics in the second and third trimesters of pregnancy is described as well as the time to fall below the clinical target concentrations for LA-CAB/RPV.

Model predictions suggest that both cabotegravir and rilpivirine could remain detectable in the maternal plasma throughout pregnancy after dosing discontinuation early in pregnancy. With lower limits of quantitation of cabotegravir and rilpivirine previously reported as 25 and 1 ng/mL [8, 47], respectively, both drugs are also predicted to remain detectable in the maternal plasma up to week 64 postmenstrual age. However, predicted plasma drug concentrations fell below the clinical target concentrations level for both drugs in all the virtual maternal population before delivery. In addition, predicted rilpivirine concentration in the cord blood at delivery was below the PA-IC_90_ (12 ng/mL). In contrast, predicted cabotegravir concentration in the cord blood was generally above the PA-IC_90_ (166 ng/mL).

The long tail-phase of LA-CAB/RPV after discontinuation is chiefly influenced by the slow-release nature of the drugs from the muscle depot where it is administered. Model predictions show that the administered drug accumulates within the muscles with each dosing until steady state is reached around 21 months. In addition, the size of the drug depot in the muscle was predicted to reach about 380 and 170% of the maintenance dose for both drugs at steady state with Q4W and Q8W dosing, respectively. It should be noted that significant differences in body composition like reduction in muscle mass due to conditions like sarcopenia might impact exposure like differences in disposition of LA-CAB/RPV observed in patients with different BMI. However, such differences in exposure could also be moderated using the right needle length and appropriate injection techniques [46].

The PBPK framework used here was not developed de novo for this manuscript, but its suitability for the present application depends on the extent to which prior qualification datasets approximate the current clinical scenario. In that regard, model support was strongest for oral and adult long-acting datasets and was further anchored by pregnancy washout data after discontinuation early in pregnancy. Simulated terminal half-lives in that setting were consistent with the available clinical observations. Nevertheless, some model comparisons approached the pre-specified acceptance boundary, and pregnancy-specific long-acting data remain limited. Accordingly, these results are best interpreted as mechanistically informed estimates for comparison of exposure patterns, threshold-crossing times, and qualitative clinical implications, rather than exact predictions of individual patient concentrations. A further methodological consideration is that a single unitary model could not be used from preconception through delivery and the postpartum period. Instead, separate but linked models were required before pregnancy, during the first trimester, during the second/third trimesters, and after delivery. We linked these stages by transferring the final residual depot amount into the next model stage because the depot acts as the dominant rate-limiting source of ongoing systemic exposure during the tail phase. This approach is mechanistically reasonable for depot-driven terminal decline, but it may still introduce discontinuity because all state variables are not propagated within a single continuous model structure. The results should therefore be interpreted with this source of uncertainty in mind. A previous study has reported that cabotegravir remained detectable up to 76 weeks after dosing discontinuation in non-pregnant adults [8]. Another study also reported the long tail-phase of LA-CAB/RPV after discontinuation in pregnancy among study participants who discontinued LA-CAB/RPV upon becoming pregnant. Though some of the study participants had not achieved steady state before the discontinuation in pregnancy, both cabotegravir and rilpivirine remained detectable until postpartum in most of the study participants. However, the drug concentrations within the cord blood at delivery was not reported [22].

The clinical implications of the pharmacokinetic tail differ between treatment and prevention settings. For individuals receiving LA-CAB/RPV for HIV treatment, discontinuation in pregnancy should, where clinically appropriate, be followed promptly by transition to an alternative suppressive regimen so that virologic control is not compromised while residual subtherapeutic concentrations persist. For individuals receiving LA-CAB for PrEP, the concern is different: declining but persistent drug exposure may eventually become insufficient for protection, while also creating a prolonged window during which resistance selection could occur if HIV acquisition takes place. Our findings therefore support early counselling regarding pregnancy planning and timely consideration of an alternative oral regimen after discontinuation. In particular, for Q8W LA-CAB used for PrEP, substitution may need to be considered before subtherapeutic concentrations are reached, which in our simulations occurred at a median of approximately 27 weeks after the last dose. However, this study did not evaluate the optimal replacement regimen or the duration of replacement therapy, both of which remain important areas for further investigation. Similar considerations may extend to other long-acting agents with prolonged terminal tails, such as lenacapavir [48]. Women of child-bearing potential who initiate long-acting injectable agents for PrEP should therefore be counselled about the likelihood of continued fetal exposure if pregnancy occurs, since fetal exposure may be difficult to avoid even when treatment is discontinued early in gestation. In addition, residual maternal plasma concentrations may persist for a considerable period after discontinuation during pregnancy or shortly before conception, with potential implications for infant exposure during breastfeeding. As postpartum physiology gradually returns towards the non-pregnant state while depot drug release continues, transfer into breast milk could contribute to infant drug exposure, although this was not investigated in the present study.

This study has several limitations. First, the entire disposition profile from preconception through delivery and into the postpartum period could not be simulated within a single unitary PBPK model. Separate but linked models were therefore used across stages, with carryover of the residual intramuscular depot amount between stages. Because this segmented workflow does not preserve all model states within one continuous structure, some discontinuity may have been introduced despite preservation of the principal source term driving the tail phase. Second, all discontinuation scenarios assumed that the last injection occurred in week 1 of pregnancy and that delivery occurred at 40 weeks for all virtual participants. In clinical practice, the timing of the final injection prior to discontinuation and the timing of delivery vary substantially; therefore, the generalisability of the predicted threshold-crossing times is limited. Third, the increase in CYP3A4 activity during pregnancy was represented as a static 1.6-fold change relative to the pre-pregnancy value, rather than as a continuously varying longitudinal function. This simplification may affect prediction accuracy for rilpivirine. Fourth, postpartum pharmacokinetics were simulated using the adult female PBPK model rather than a dedicated postpartum model. Consequently, rapid reversal of pregnancy physiology after delivery and transfer into breast milk were not represented explicitly. Infant exposure via breastfeeding therefore remains uncharacterised in the present work. Finally, the present virtual population was based on previously reported Caucasian demographic and organ-weight parameters. Although pregnancy-related physiological changes and body-weight increases were implemented dynamically, the framework may not fully represent populations with different body composition, anthropometry, or ethnic background. This is relevant because depot kinetics and exposure to long-acting injectables may vary with factors such as BMI, muscle mass, and injection characteristics. Extrapolation beyond the simulated population should therefore be made cautiously.

## Conclusions

The discontinuation of LA-CAB/RPV shortly before or early in pregnancy is likely to result in fetal exposure to the discontinued drug throughout gestation. Also, the benefit-risk considerations of switching to an oral antiretroviral regimen need to be considered carefully. Potentially, infant exposure via breastfeeding could also occur as residual drug concentrations are likely to remain detectable in maternal plasma postpartum. Women discontinuing LA-CAB for PrEP might need to switch to oral PrEP to reduce the risk of the development of drug resistance. Further studies are required to investigate the necessary duration for an oral PrEP after discontinuing LA-CAB.

## Supplementary Information

Below is the link to the electronic supplementary material.

Supplementary File 1 (PDF 272 KB)

## Data Availability

Data support the findings of this study are available from the corresponding author upon reasonable request.
