# Supplementary material for: Modelling the tail-phase pharmacokinetics of long-acting cabotegravir and rilpivirine from early pregnancy to postpartum at steady state
Source: J Pharmacokinet Pharmacodyn. 2026 Sep 23;53(6):55. doi: 10.1007/s10928-026-10065-4 (PMC13601093; doi:10.1007/s10928-026-10065-4)
Supplement: Supplementary file 1 [file 10928_2026_10065_MOESM1_ESM.pdf]

Supplementary Information

Table S1: Input drug parameters for the CAB, RAL and RPV models reproduced from Atoyebi et al. [1]

**Drug parameter**	**CAB**	**RAL**	**RPV**
Molecular weight	405	444	366
pKa	10.04 [2]	6.67 [3]	3.26 [2]
R	0.441 [2]	0.6 [3]	0.67 [2]
Log $P_{o:w}$	1.04 [2]	0.58 [3]	4.32 [2]
f_{up}	0.007 [2]	0.17 [3]	0.003 [2]
HBD	2 [2]		
PSA	99.2 [2]		
Caco-2 P_{app} x 10^{-6} (cm/s)		6.6 [3]	12 [2]
CYP3A4 CL_{int} (µL/min/pmol)			2.04 [2]
UGT1A1 CL_{int} (µL/min/mg)	4.5 [2]	12.4[a] [3]	
UGT1A9 CL_{int} (µL/min/mg)	2.2 [2]		
IM drug release rate[b] (h^{-1})	3.406 x 10^{-4}		4.5 x 10^{-4}
Drug diffusion rate constant across placenta	0.028[c]	0.015[b]	0.027[c]

CAB – cabotegravir, RAL – raltegravir, RPV - rilpivirine, pKa - acid dissociation constant, R - blood-to-plasma drug ratio, Log $P_{o:w}$ - partition coefficient between octanol and water, f_{up} - fraction of drug unbound in plasma, HBD - number of hydrogen bond donors, PSA - polar surface area, P_{app} - apparent permeability coefficient, Cl_{int} - intrinsic clearance, CYP - cytochrome P450, UGT - uridine diphosphate-glucuronosyltransferase.

[a] µL/min/10^6 hepatocytes.

[b] value fitted in the model using available clinical PK data.

[c] value derived using the diffusion constant and Papp of RAL alongside the Papp of the drug.

Table S2: Key pregnancy-induced anatomical, physiological, and metabolic changes implemented in the pregnancy model reproduced from Atoyebi et al. [1]

**Parameter**	**Equation**	**Reference**
Body weight (kg)	Body weight = $61.1 + 0.2409GA + 0.0038GA^2$	[4]
Cardiac output (L/h)	Cardiac output = $301 + 5.916GA - 0.088GA^2$	[4]
Plasma proteins (g/L)	Plasma proteins = $69.7 + 0.2085GA - 0.0305GA^2 + 0.0006GA^3$	[4]
CYP3A4 enzyme activity	CYP3A4 activity = X 1.6	[5]

CYP - cytochrome P450, GA – gestational age in pregnancy (weeks).

Table S3: Validation of adult PBPK model for oral PK of CAB, RPV and RAL (simulated vs observed) reproduced from Atoyebi et al. [1]

PK parameter	Observed	Simulated	AAFE
30 mg CAB (single dose)* [6]			
C_{max} (µg/ml)	3.61	3.07	1.18
$AUC_{0\text{-}inf}$ (µg.h/ml)	146	98.2	1.49
30 mg CAB (repeated doses)* [7]			
C_{trough} (µg/ml)	4.9	4.6	1.06
C_{max} (µg/ml)	8.3	7.8	1.06
$AUC_{0\text{-}24}$ (µg.h/ml)	147	154	1.05
25 mg RPV (repeated doses)^ [8]			
C_{min} (ng/mL)	89.85	68.9	1.30
C_{max} (ng/mL)	203.8	219.8	1.08
$AUC_{0\text{-}24}$ (ng.h/mL)	2589	3305	1.28
25 mg RPV (repeated doses)^ [9]			
C_{trough} (ng/mL)	67.3	62.2	1.08
C_{max} (ng/mL)	180.9	189.60	1.05
$AUC_{0\text{-}24}$ (ng.h/mL)	2528	2895.7	1.15
25 mg RPV (repeated doses)* [10]			
C_{trough} (ng/mL)	74.5	62.7	1.19
C_{max} (ng/mL)	148	176.01	1.19
$AUC_{0\text{-}24}$ (ng.h/mL)	2227	2779.8	1.25
25 mg RPV (repeated doses)* [10]			
C_{trough} (ng/mL)	87.4	66.7	1.31
C_{max} (ng/mL)	171	177.68	1.04
$AUC_{0\text{-}24}$ (ng.h/mL)	2473	2860.3	1.16
150 mg RPV (repeated doses)^ [11]			
C_{trough} (ng/mL)	478	400.6	1.19
C_{max} (ng/mL)	1123	1296	1.15
$AUC_{0\text{-}24}$ (ng.h/mL)	16051	19398	1.21
400 mg RAL (repeated doses)* [12]			
C_{trough} (ng/ml)	79.7	156	1.96
C_{max} (ng/ml)	3035	1706	1.78
$AUC_{0\text{-}12}$ (ng.h/ml)	11600	10244	1.13

*Data presented are Geometric mean values, ^Data presented are arithmetic mean values,
AAFE – absolute average fold error, CAB – cabotegravir, RAL – raltegravir, RPV - rilpivirine, C_{trough} – plasma concentration at the end of the dosing interval, C_{max} – maximum plasma concentration, $AUC_{0\text{-}inf}$ – area under the plasma plasma concentration time curve till infinity, $AUC_{0\text{-}12}$ – area under the plasma plasma concentration time curve within 12 hours.

Table S4: Validation of adult PBPK model for PK of LAI CAB and LAI RPV (simulated vs observed) reproduced from Atoyebi et al. [1]

Regimen	**C_τ (µg/ml)**			**C_{max} (µg/ml)**			**$AUC_{0-\tau}$ (µg.h/ml)**		
	Observed*	Simulated	AAFE	Observed*	Simulated	AAFE	Observed*	Simulated	AAFE
CAB									
800 mg IM + 200 mg IM monthly	1.61	1.68	1.04	2.2	2.0	1.09	1242	1255	1.01
800 mg IM + 400 mg IM monthly	3.27	2.71	1.21	4.4	3.3	1.35	2473	2020	1.22
800 mg IM quarterly	1.1	1.4	1.26	3.3	2.6	1.27	4467	3947	1.27
	**C_τ (ng/ml)**			**C_{max} (ng/ml)**			**$AUC_{0-\tau}$ (ng.h/ml)**		
	Observed*	Simulated	AAFE	Observed*	Simulated	AAFE	Observed*	Simulated	AAFE
RPV									
1200 mg IM + 600 mg IM monthly	78.9	109.4	1.39	126	143	1.13	63656	84685	1.33
1200 mg IM + 900 mg IM monthly	79.1	132.8	1.68	168	173	1.03	74420	102489	1.38

Data presented as geometric mean values. *Clinical data observed by Spreen et al 2014 [7]. C_τ – plasma concentration at the end of the dosing interval, C_{max} – maximum plasma concentration, $AUC_{0-\tau}$ – area under the plasma concentration time curve within the dosing interval, IM – intramuscular, RAL – raltegravir, RPV - rilpivirine, CAB – cabotegravir, LAI – long-acting injectable.

Table S5: Validation of pregnancy PBPK model for RAL, CAB and RPV in pregnant women (simulated vs observed) reproduced from Atoyebi et al. [1]

PK parameter	Observed	Simulated	AAFE
Oral RAL			
2nd trimester*a			
C_{12} (ng/ml)	62.1	36.5	1.70
C_{max} (ng/ml)	2250	1127	1.99
AUC_{0-12} (ng.h/ml)	6600	5461	1.21
3rd trimester*a			
C_{12} (ng/ml)	64	34	1.88
C_{max} (ng/ml)	1770	1088	1.63
AUC_{0-12} (ng.h/ml)	5400	5263	1.03
Delivery*a			
C:M ratio	1.5	1.25	1.20
Cord blood (ng/ml)	154	150	1.02
Maternal blood (ng/ml)	140	162	1.15
Oral RPV			
2nd trimester#b			
C_{min} (ng/ml)	54.3	42.7	1.27
C_{max} (ng/ml)	121	191	1.58
AUC_{0-24} (ng.h/ml)	1792	2719	1.52
3rd trimester#b			
C_{min} (ng/ml)	52.9	40.9	1.29
C_{max} (ng/ml)	123	189	1.54
AUC_{0-24} (ng.h/ml)	1762	2675	1.52
3rd trimester^c			
C_{min} (ng/ml)	50	41	1.21
C_{max} (ng/ml)	110	190	1.73
AUC_{0-24} (ng.h/ml)	1710	2691	1.57
Delivery*c			
C:M ratio	0.5	0.81	1.61
LAI RPV#d			
Terminal $t_{1/2}$ (weeks)	10.7	8.92	1.21
LAI CAB#d			
Terminal $t_{1/2}$ (weeks)	8.99	10.0	1.12

*Median values. ^Geometric mean values. #Arithmetic mean values. RAL – raltegravir, RPV - rilpivirine, CAB – cabotegravir, C:M ratio – cord-to-maternal blood concentration ratio at delivery, C_{12} – plasma concentration 12 hrs after dose administration, C_{min} – minimum plasma concentration, C_{max} – maximum plasma concentration, AUC_{0-inf} – area under the plasma plasma concentration time curve till infinity, AUC_{0-12} – area under the plasma

plasma concentration time curve within 12 hours, AUC_{0-24} – area under the plasma plasma concentration time curve within 24 hours, Terminal $t_{1/2}$ – terminal half-life.

[a]Watts et al 2014 [12]; [b]Blonk et al 2015 [13]; [c]Schalkwijk et al 2017 [14]; [d]Patel et al 2023 [15]

Reference

1. Atoyebi S, Bunglawala F, Cottura N, Grañana-Castillo S, Montanha MC, Olagunju A, Siccardi M, Waitt C (2025) Physiologically-based pharmacokinetic modelling of long-acting injectable cabotegravir and rilpivirine in pregnancy. British Journal of Clinical Pharmacology 91 (4):989-1002. doi:https://doi.org/10.1111/bcp.16006
2. Rajoli RKR, Back DJ, Rannard S, Meyers CF, Flexner C, Owen A, Siccardi M (2018) In Silico Dose Prediction for Long-Acting Rilpivirine and Cabotegravir Administration to Children and Adolescents. Clinical Pharmacokinetics 57 (2):255-266. doi:10.1007/s40262-017-0557-x
3. Rajoli RKR, Back DJ, Rannard S, Freel Meyers CL, Flexner C, Owen A, Siccardi M (2015) Physiologically Based Pharmacokinetic Modelling to Inform Development of Intramuscular Long-Acting Nanoformulations for HIV. Clinical Pharmacokinetics 54 (6):639-650. doi:10.1007/s40262-014-0227-1
4. Abduljalil K, Furness P, Johnson TN, Rostami-Hodjegan A, Soltani H (2012) Anatomical, physiological and metabolic changes with gestational age during normal pregnancy: a database for parameters required in physiologically based pharmacokinetic modelling. Clin Pharmacokinet 51 (6):365-396. doi:10.2165/11597440-000000000-00000
5. Dallmann A, Ince I, Coboeken K, Eissing T, Hempel G (2018) A Physiologically Based Pharmacokinetic Model for Pregnant Women to Predict the Pharmacokinetics of Drugs Metabolized Via Several Enzymatic Pathways. Clinical pharmacokinetics 57 (6):749-768. doi:10.1007/s40262-017-0594-5
6. Ford SL, Sutton K, Lou Y, Zhang Z, Tenorio A, Trezza C, Patel P, Spreen W (2017) Effect of Rifampin on the Single-Dose Pharmacokinetics of Oral Cabotegravir in Healthy Subjects. Antimicrob Agents Chemother 61 (10). doi:10.1128/aac.00487-17
7. Spreen W, Williams P, Margolis D, Ford SL, Crauwels H, Lou Y, Gould E, Stevens M, Piscitelli S (2014) Pharmacokinetics, safety, and tolerability with repeat doses of GSK1265744 and rilpivirine (TMC278) long-acting nanosuspensions in healthy adults. J Acquir Immune Defic Syndr 67 (5):487-492. doi:10.1097/qai.0000000000000365
8. Center for Drug Evaluation and Research (2011) Clinical Pharmacology and Biopharmaceutics Review(s). Addendum to Ondqa Biopharmaceutics review
9. Crauwels H, Vingerhoets J, Ryan R, Witek J, Anderson D (2012) Pharmacokinetic Parameters of Once-Daily Rilpivirine following Administration of Efavirenz in Healthy Subjects. Antiviral Therapy 17 (3):439-446. doi:10.3851/imp1959
10. Ford SL, Gould E, Chen S, Margolis D, Spreen W, Crauwels H, Piscitelli S (2013) Lack of pharmacokinetic interaction between rilpivirine and integrase inhibitors dolutegravir and GSK1265744. Antimicrobial agents and chemotherapy 57 (11):5472-5477. doi:10.1128/AAC.01235-13
11. Rajoli RKR, Curley P, Chiong J, Back D, Flexner C, Owen A, Siccardi M (2019) Predicting Drug-Drug Interactions Between Rifampicin and Long-Acting Cabotegravir and Rilpivirine Using Physiologically Based Pharmacokinetic Modeling. J Infect Dis 219 (11):1735-1742. doi:10.1093/infdis/jiy726
12. Watts DH, Stek A, Best BM, Wang J, Capparelli EV, Cressey TR, Aweeka F, Lizak P, Kreitchmann R, Burchett SK, Shapiro DE, Hawkins E, Smith E, Mirochnick M (2014) Raltegravir pharmacokinetics during pregnancy. J Acquir Immune Defic Syndr 67 (4):375-381. doi:10.1097/qai.0000000000000318
13. Blonk MI, Colbers AP, Hidalgo-Tenorio C, Kabeya K, Weizsäcker K, Haberl AE, Moltó J, Hawkins DA, van der Ende ME, Gingelmaier A, Taylor GP, Ivanovic J, Giaquinto C, Burger DM (2015) Raltegravir in HIV-1-Infected Pregnant Women: Pharmacokinetics, Safety, and Efficacy. Clin Infect Dis 61 (5):809-816. doi:10.1093/cid/civ366
14. Schalkwijk S, Colbers A, Konopnicki D, Gingelmaier A, Lambert J, van der Ende M, Moltó J, Burger D, Network ftPondaaiH-ipw (2017) Lowered Rilpivirine Exposure During the Third Trimester of Pregnancy in Human Immunodeficiency Virus Type 1–Infected Women. Clinical Infectious Diseases 65 (8):1335-1341. doi:10.1093/cid/cix534
15. Patel P, Ford SL, Baker M, Meyer C, Garside L, D'Amico R, Van Solingen-Ristea R, Crauwels H, Polli JW, Seal C, Yagüe Muñoz I, Thiagarajah S, Birmingham E, Spreen WR, Baugh B, van Wyk J, Vannappagari V (2023) Pregnancy outcomes and pharmacokinetics in pregnant women living with HIV exposed to long-acting cabotegravir and rilpivirine in clinical trials. HIV Medicine 24 (5):568-579. doi:https://doi.org/10.1111/hiv.13439
